# A New Technique for Improving Visualization of Mucosal Lesions During Endoscopic Photodynamic Therapy

**DOI:** 10.1155/DTE.6.147

**Published:** 2000

**Authors:** Seishiro Mimura, Hiroyuki Narahara, Takashi Murakami, Toru Otani, Toru Hirano, Kenji Suzuki

**Affiliations:** 1 Department of Gastroenterology Osaka Medical Center for Cancer and Cardiovascular Diseases 1-3-3 Nakamichi, Higashinari-ku Osaka 537-8511 Japan; 2 photon Medical Research Center Hamamatsu University School of Medicine Japan; 3 Hamamatsu Photonics K.K. Japan

## Abstract

A new device consisting of a conventional fiberscope and a new TV system (model OTV-S5, Olympus Optical Co., Tokyo, Japan) has been developed to achieve accurate irradiation of laser light in photodynamic therapy for gastric cancer. This model has high resolution and sensitivity, and its signal can be transmitted by red, green and blue. In front of the CCD we inserted a special interference filter which has specific absorption of red light with 2.3% transmissivity at a 630 nm wavelength and a 50 nm absorption band of full width at half maximum. The average transmittance in the visible region, except for at 630 nm, was 90%. A neutral density filter with 16% transmittance was added to adjust to the sensitivity of the CCD. The device makes it possible to perform accurate irradiation, because we can observe both the lesion and the laser spot on a monitor in original colors during irradiation.


*Diagnostic and Therapeutic Endoscopy*, Vol. 6, pp. 147–152
Reprints available directly from the publisher
Photocopying permitted by license only

© 2000 OPA (Overseas Publishers Association) N.V.
Published by license under
the Harwood Academic Publishers imprint,
part of The Gordon and Breach Publishing Group.
Printed in Malaysia.

### A New Technique for Improving Visualization of Mucosal Lesions during Endoscopic Photodynamic Therapy

SEISHIRO MIMURA[a,*], HIROYUKI NARAHARA[a], TAKASHI MURAKAMI[a], TORU OTANI[a], TORU HIRANO[b] and KENJI SUZUKI[c]

[a]*Department of Gastroenterology, Osaka Medical Center for Cancer and Cardiovascular Diseases, 1-3-3 Nakamichi, Higashinari-ku, Osaka 537-8511, Japan;* [b]*Photon Medical Research Center, Hamamatsu University, School of Medicine, Japan;* [c]*Hamamatsu Photonics K.K., Japan*

*(Received 5 January 2000; In final form.15 February 2000)*

**A new device consisting of a conventional fiberscope and a new TV system (model OTV-S5, Olympus Optical Co., Tokyo, Japan) has been developed to achieve accurate irradiation of laser light in photodynamic therapy for gastric cancer. This model has high resolution and sensitivity, and its signal can be transmitted by red, green and blue. In front of the CCD we inserted a special interference filter which has specific absorption of red light with 2.3% transmissivity at a 630 nm wavelength and a 50 nm absorption band of full width at half maximum. The average transmittance in the visible region, except for at 630 nm, was 90%. A neutral density filter with 16% transmittance was added to adjust to the sensitivity of the CCD. The device makes it possible to perform accurate irradiation, because we can observe both the lesion and the laser spot on a monitor in original colors during irradiation.**

*Keywords:* Device for photoradiation, Endoscopic treatment, Gastric cancer, Photodynamic therapy

### INTRODUCTION

Photodynamic therapy (PDT) has been proved to be a safe and promising alternative for the treatment of patients with early stage cancer of the lung [1,2], esophagus [3,4] and stomach [5] who are poor risks for surgery, and it was approved by the Ministry of Health and Welfare of Japan in 1994 [6]. However, it is very difficult to accurately irradiate the lesion with enough energy, because it is difficult to clearly observe both the cancerous lesion, and also the boundary of the laser spot during irradiation. To achieve accurate irradiation of laser light, it is necessary to improve the endoscopic device for PDT. Initially we placed a green filter [7] on the eyepiece of the fiberscope to protect the naked eye from being dazzled during photoradiation. Later, we also inserted a green filter in front of the CCD-camera in an OES-TV system with an NTSC signal. The red spot of laser light could be observed clearly, but the mucosal image turned green and the boundary between cancer and surrounding mucosa became

* Corresponding author. Tel.:81 6 6972 1181. Fax: 81 6 6981 4067.

unclear. For observing mucosal change at a pause of irradiation, we have to remove the filter, and insert it again before restarting irradiation. In the former study [8] we employed a new OES-TV system (model OTV-S5, Olympus Optical Co., Tokyo, Japan) that has high resolution and sensitivity. We also employed an interference filter instead of a green filter and tried to add several grades of a neutral density filter to adapt to the sensitivity of the CCD. However, these filters were not enough to absorb the red laser beam. In the present study we therefore, designed a new interference filter with a deeper absorption band at a wavelength of 630 nm [9]. In this paper we present not only the new device but also historical instruments with some clinical cases.

### MATERIALS AND METHODS

The light source for irradiation in PDT was an excimer dye laser [10] (EDL, Hamamatsu Photonics, Hamamatsu, Japan), which has been improved to possess the following characteristics: wavelength, 630 nm; pulse energy, 4 mJ; peak power, 400 kW; pulse width, 10 ns; frequency of repetition, 30, 40 or 80 Hz (80 Hz was used in this trial); average output, 320 mW. The laser beam is condensed and transmitted through a 400-μm core diameter quartz fiber. When the distance between the tumor surface and the simple cut fiber tip is 3.2 cm and the diversion angle of the laser beam is 20°, the irradiation area is approximately 1 $cm^2$. The new device consists of a conventional side-viewing fiberscope (model OES GF-20, Olympus) and an OES-TV system (model OTV-S5, Olympus). It has a high resolution with 410,000 picture elements and more than 600 lines and also has a high sensitivity which allows it to form an image at 1.5 lx with a maximum gain. The signals can be transmitted by red, green and blue (RGB). This system has an automatic gain controller in order to be adjustable to two kinds of methods for measuring radiance. One measures the average radiance and the other measures the peak, and they are instantaneously interchangeable. In front of the CCD we inserted a new interference filter which had specific absorption of red light, and we added a neutral density filter of 0.8 density, i.e. 16.4% transmittance as shown in Fig. 1. The

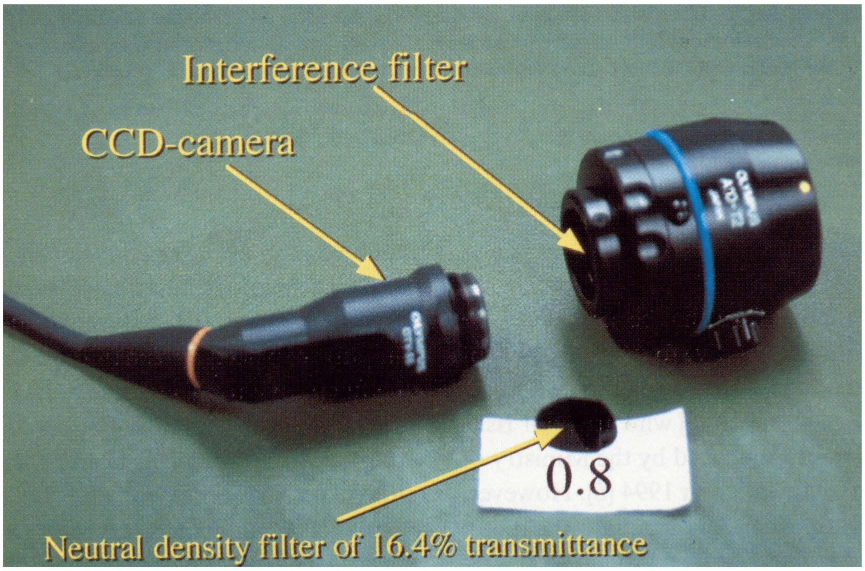

FIGURE 1 *CCD-camera and filters*. The larger part on the right is a connector between an eyepiece of a fiberscope and a CCD-camera, in which the interference filter has been inserted. The smaller part on the left is the CCD-camera. A neutral density filter of 16.4% transmittance appears between them.

characteristics of the interference filter show 2.3% transmissivity at a 630 nm wavelength and a 50 nm absorption band of full width at half maximum, in spite of 90% average transmittance in the visible region, except for at 630 nm as shown in Fig. 2.

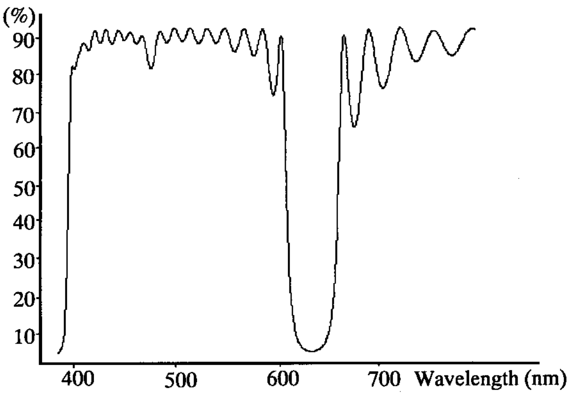

FIGURE 2 *Spectral transmissivity of the interference filter.* The interference filter has a specific absorption band of red light with 2.3% transmissivity at a wavelength of 630 nm and a 50 nm absorption band of full width at half maximum, while the average transmittance in the visible region, except for at 630 nm, is 90%. For practical use we added a neutral density filter of 0.8 density, i.e. 16.4% transmittance to adapt to the sensitivity of the CCD.

### RESULTS

Before and during pauses of irradiation the mucosal image could be observed on a monitor as clearly as on a conventional fiberscope-TV system as shown in Fig. 3(a). Deviation of color balance of gastric mucosa was negligible, while gray chart observation sensitivity deviated slightly towards blue because of red light reduction by the interference filter, as shown in Fig. 4(a). During irradiation the mucosal image did not change (Fig. 3(b)), while the image of the gray chart was adjusted to an original gray, because reflection of the red laser light compensated the deviation towards blue as shown in Fig. 4(b). The laser beam was observed as a red spot when the irradiation was being performed at a long or medium distance between the fiber tip and the mucosa, while at too short a distance the laser spot turned to white i.e. a highlight. In this way we could observe the mucosa clearly during the entire course of irradiation and pauses without inserting or removing of the filter.

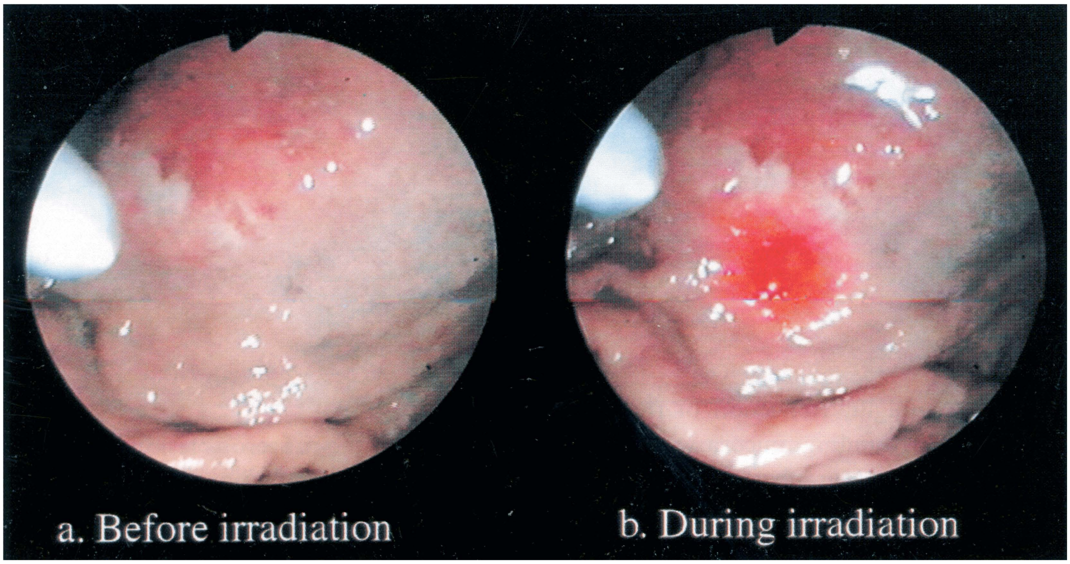

FIGURE 3 The endoscopic mucosal images in PDT obtained by the new device. The deviation of color balance is negligible both before and during irradiation.

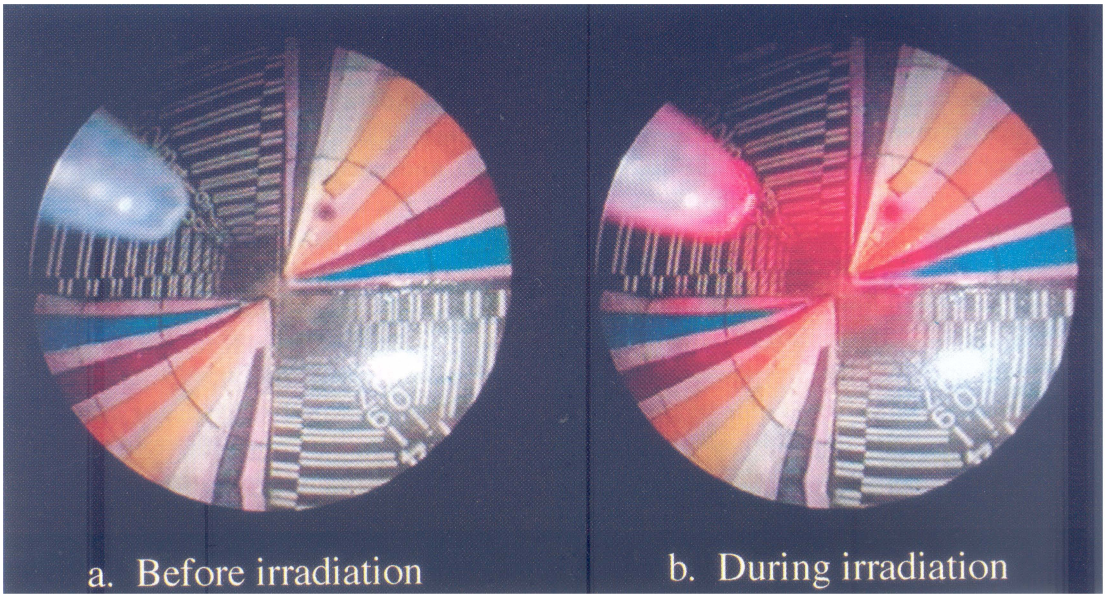

FIGURE 4 The endoscopic images of a color chart obtained by the new device. The color balance before irradiation deviated slightly toward blue, while that during irradiation was adjusted to an original gray.

### DISCUSSION

In the early years of PDT around 1980s, we placed a green filter on the eyepiece of the fiberscope to protect the naked eye from being dazzled by reflection of the laser light. Later, we also inserted a green filter in front of the CCD-camera in the fiberscope-TV system as related above. Figure 5 shows spectral transmissivity of each green filter, SP-15 is for observation and photography, and SP-19 for the CCD. The transmissivity at 630 nm wavelength was 2% in SP-15, and 17% in SP-19. Figure 6 shows one frame from a VTR obtained without filter, before irradiation and during irradiation. Before irradiation the image of mucosa was clear, during irradiation however, a laser spot appeared white. This means excessive irradiation and is called a highlight. Reflection of the laser beam tinged the surrounding mucosa with a rosy flush. In Fig. 7, each photograph was obtained with a green SP-19 filter in front of the CCD, before irradiation and during irradiation. The red spot of the laser light could be observed clearly, but the mucosal image turned green and dark. Thus, the

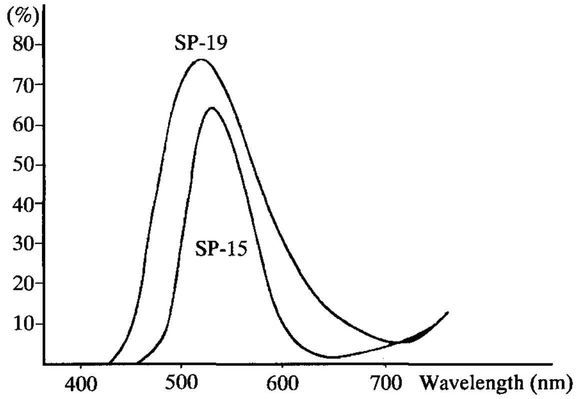

FIGURE 5 *Spectral transmissivity of the green filters.* A green filter (model SP-15) is made of acetate for sensitivity tests of X-ray film, with a peak transmittance of 63% with a wavelength of 530 nm. The transmissivity at 630 nm is 2%. A green filter (model SP-19) is one of the filters for separating three primaries, i.e. RGB, with a peak transmittance of 75% with a wavelength of 520 nm and 17% at 630 nm.

boundary of the cancerous lesion became unclear, not only during irradiation but also during pauses of irradiation. We therefore, introduced an interference filter to reduce the red of the laser light while keeping the original color of the gastric mucosa. Absorption of red light by the filter of the

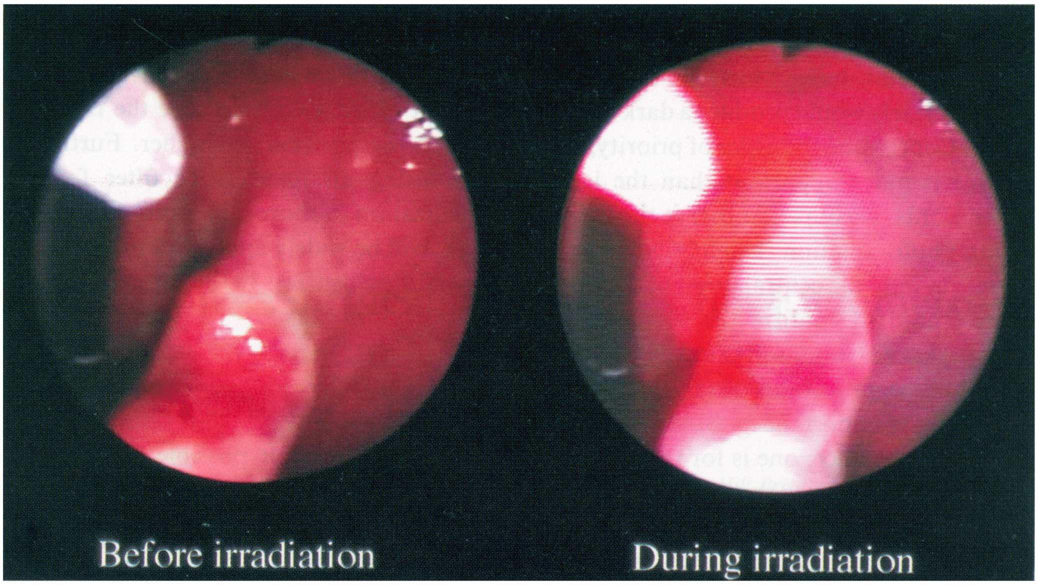

FIGURE 6 The endoscopic mucosal images in PDT obtained by the old device without a filter, before irradiation and during irradiation.

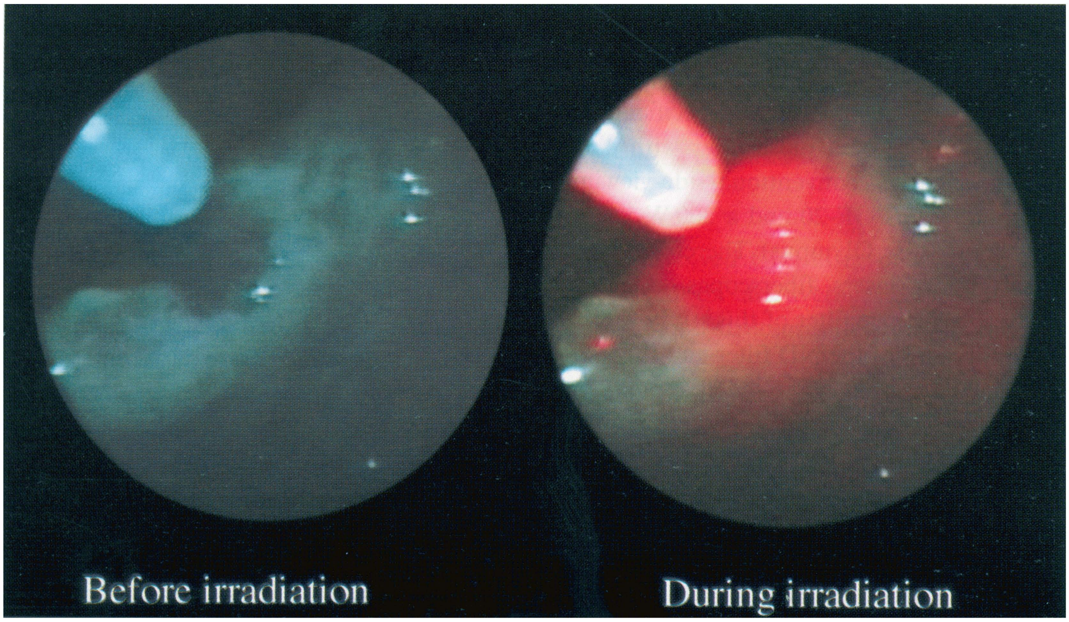

FIGURE 7 The endoscopic mucosal images in PDT obtained by the old device with a green filter SP-19 in front of the CCD-camera, before irradiation and during irradiation.

former study was insufficient, therefore, an interference filter with deeper absorption of red light was made and good results were obtained. However, in spite of excellent absorption of laser light, it was impossible in the strict sense of the word, to observe the lesion and the laser spot in original colors simultaneously. When absorption of the laser light is almost total, although the mucosal image was shown in original color, the radiance of the laser spot would be too low to observe clearly. On the other hand, conditions under which the laser spot can be observed clearly would produce a dark image of the mucosa. Concerning the order of priority, the mucosal image is more important than the laser spot. In other words, we hope to observe the lesion clearly during photoradiation. Namely, we would expect to confirm only the central point of the laser spot, while we need to confirm the size and the brightness of the laser at initiation of irradiation and when moving the beam to other areas. Therefore, for practical use it is important to satisfy two conditions for optimal balance; one is for the mucosa, and the other is for the laser spot. The condition for mucosa was obtained by measuring the average radiance. The condition for the laser spot was obtained by decreasing the radiance of the xenon arc and measuring the peak radiance. In this way the former enables us to observe the image of the mucosa clearly, while the laser spot become unclear. The latter enables us to observe the laser spot clearly, while the image of mucosa become dark. In the present study, selection of filter specifications was based on the use of a pulse laser with 4 mJ pulse energy and 80 Hz, a conventional side-viewing fiberscope model OES GF-20 and a 300 W xenon arc (model CLV-U20D, Olympus). Therefore, when using other types of lasers, fiberscopes or lamps, although the interference filter of this study will be useful, several grades of the ND filter and the level of gain controller should be tested to obtain an optimal balance. Moreover, the interference filter may be useful for other types of fiberscope-TV system. Although the filter is not on sale, it is available when purchasing the laser equipment of the company. The spectral transmissivity in Fig. 2 was measured in the condition of the light in parallel. At the position where the filter was inserted in this study, i.e. in front of the CCD, the light is not necessarily parallel. Generally speaking, transmissivity differs greatly depending on the angle of incidence into the interference filter: oblique light is easier to transmit. Therefore, the filter effectiveness in front of the CCD is slightly weaker (i.e. transmissivity is higher) than the measured value in the figure. Moreover, when using it on an eyepiece, it will be not so effective as when inserted in front of the CCD, because the rates of oblique light at the position are higher. Furthermore, the idea, using an interference filter for improving visualization of mucosal lesions during endoscopic PDT, will be widely applied to the PDT employing new photosensitizers in the future.

### CONCLUSION

The device makes it possible to achieve accurate irradiation, because we can observe both the lesion and the laser spot in original colors on a monitor during irradiation.

### *References*

[1] Hayata, Y., Kato, H., Konaka, C. *et al.* Hematoporphyrin derivative and laser photoradiation in the treatment of lung cancer. *Chest* 1982; **81**: 269–277.

[2] Kato, H., Horai, T., Furuse, K. *et al.* Photodynamic therapy for cancers: a clinical trial of porfimer sodium in Japan. *Jpn. J. Cancer. Res.* 1993; **84**: 1209–1214.

[3] Mimura, S., Otani, T. and Okuda, S. Photodynamic therapy for superficial esophageal cancer using an excimer dye laser. *Diagn. Ther. Endosc.* 1994; **1**: 99–105.

[4] Sibille, A., Lambert, R., Souquet, J.C. *et al.* Long-term survival after photodynamic therapy for esophageal cancer. *Gastroenterology* 1995; **108**: 337–344.

[5] Mimura, S., Ito, Y., Nagayo, T. *et al.* Cooperative clinical trial of photodynamic therapy with Photofrin II and excimer dye laser for early gastric cancer. *Lasers Surg. Med.* 1996; **19**: 168–172.

[6] Hayata, Y., Kato, H., Furuse, K. *et al.* Photodynamic therapy of 168 early stage cancers of the lung and oesophagus: a Japanese multi-centre study. *Lasers Med. Sci.* 1996; **11**: 255–259.

[7] Fuji Film. Fuji film optical filter manual, 1993 (in Japanese).

[8] Mimura, S., Narahara, H., Otani, T. *et al.* Development of a new device for endoscopic photoradiation in PDT, in *7th Japanese Chapter Anniversary Meeting of International Photodynamic Association (JCIPA)*, Nishisaka, T. (Ed.) *Proc. Recent Progress of Photodynamic Therapy*, 1997; pp. 81–84.

[9] Mimura, S., Narahara, H., Otani, T. *et al.* Development of a Device for Endoscopic Photoradiation in Photodynamic Therapy, in *7th International Photodynamic Association Biennial Meeting*, Patrice, T. (Ed.) *Proc.* 1998; RC193.

[10] Hirano, T., Ishizuka, M., Suzuki, K. *et al.* Photodynamic cancer diagnosis and treatment system consisting of pulsed lasers and an endoscopic spectro-image analyzer. *Lasers Life Sci.*, 1989; **3**: 99–116.

